# Sexual behaviour and HIV/sexually transmitted infection risk behaviours in the general population of Slovenia, a low HIV prevalence country in central Europe

**DOI:** 10.1136/sti.2008.034256

**Published:** 2008-12-05

**Authors:** I Klavs, L C Rodrigues, K Wellings, H A Weiss, R Hayes

**Affiliations:** 1 Institute of Public Health of the Republic of Slovenia, Ljubljana, Slovenia; 2 London School of Hygiene and Tropical Medicine, London, UK

## Abstract

**Objectives:**

To describe sexual and HIV/sexually transmitted infection (STI) risk behaviours in Slovenia.

**Methods:**

A nationally representative cross-sectional survey of the general population aged 18–49 years in 1999–2001 was conducted. The data were collected by face-to-face interviews and anonymous self-administered questionnaires. Statistical methods for complex survey data were used.

**Results:**

849 men and 903 women were interviewed. In the past 5 years, both men and women reported a median of one heterosexual partner (means 3.2, 1.5, respectively), concurrent heterosexual partnerships were reported by 24.4% of men and 8.2% of women, heterosexual sex with non-Slovenian partners by 12.6% of men and 12.2% of women, forced sex by 4.8% of women, paid heterosexual sex by 2.6% of men, sex with another man by 0.6% of men and heterosexual sex with an injecting drug user by 1.2% of men and 1.3% of women. In the past year, 22.7% of men and 9.5% of women reported forming at least one new heterosexual partnership. The mean numbers of episodes of heterosexual sex in the previous 4 weeks were 6.1 for men and 6.0 for women. Consistent and inconsistent condom use was reported more frequently among men reporting multiple female partners and those not married or cohabiting.

**Conclusions:**

Recent patterns of reported sexual behaviour are consistent with a low risk of HIV and STI transmission in Slovenia. The results will inform Slovenian sexual health policies including HIV/STI prevention, and are particularly valuable because population-based data on HIV/STI risk behaviour have not previously been available in low HIV prevalence countries of central Europe.

Slovenia is one of the low HIV prevalence countries of central Europe with less than one infected individual per 1000 population.^1^ Men who have sex with men (MSM) have the highest prevalence, but this remains consistently below 5%.^1^ ^2^ Reported incidence rates of sexually transmitted infections (STI) are low, but are known to underestimate the true burden of STI.^3^ In the year 2000, in a probability sample of 18–49-year-old Slovenians, 5.5% of men and 5.1% of women reported ever being diagnosed with an STI^4^ and we estimated the national prevalence of genital *Chlamydia trachomatis* infection among 18–24 year olds as 4.1%, indicating gaps in prevention, diagnosis and treatment.^5^

Reliable data on patterns of sexual behaviour are needed to inform sexual and reproductive health policies, including those for the prevention of HIV and STI. In the late 1980s and early 1990s, almost all western European countries, and many other developed and developing countries, conducted nationally representative sexual behaviour surveys.^6^^–^12 In 1999–2001, the Institute of Public Health of the Republic of Slovenia conducted the first Slovenian national Sexual Lifestyles, Attitudes and Health Survey (SLAHS, 2000). In this paper we report on sexual partnerships and practices, HIV/STI risk behaviours and recent condom use.

## METHODS

We used stratified two-stage probability sampling of 18–49-year-old Slovenians based on the central population register. Data were collected between November 1999 and February 2001 at respondents’ homes by face-to-face interviews and anonymous self-administered pencil and paper questionnaires. Questionnaires adapted from the British National Survey of Sexual Attitudes and Lifestyles (NATSAL) conducted in 1990^13^ were similar to those used in the second British survey (NATSAL, 2000).^14^

Weights were computed to adjust for differences in survey response and any remaining differences between the achieved sample and available Slovenian population estimates according to statistical regions, types of communities, gender and age groups, based on central population register data for the year 2000.

Response rates were calculated from unweighted data. Other analyses were conducted using statistical methods for complex survey data to account for stratification, clustering and weighting, using STATA version 7.0. Age at interview was grouped as 18–24, 25–34 and 35–49 years, corresponding almost exactly with birth cohorts 1975–82, 1965–74 and 1950–64. Weighted proportions with 95% CI were computed for different reported behaviours, stratified by gender, age group and marital status when appropriate. Tests for heterogeneity of proportions were computed using the Pearson χ^2^ test based on F statistics using a second-order Rao and Scott correction accounting for the survey design.

Ethics approval was obtained from the ethics committees of the Ministry of Health of the Republic of Slovenia and the London School of Hygiene and Tropical Medicine.

## RESULTS

We interviewed 1752 individuals aged 18–49 years. The overall survey response rate was 67.0%. Table 1 shows the distribution of numbers of heterosexual (opposite sex) partners reported by men and women during their lifetime, in the past 5 years and in the past year by age group. Most men (79.5%; 95% CI 76.4% to 82.3%) reported more than one lifetime female partner and 27.7% (95% CI 24.4% to 31.2%) reported 10 or more. Significantly fewer women (57.9%; 95% CI 54.2% to 61.4%) reported more than one lifetime male partner and only 5.6% (95% CI 4.1% to 7.5%) reported 10 or more. The mean numbers of lifetime heterosexual partners (8.3 for men, 3.2 for women; p<0.001) were influenced by those reporting very large numbers. Older men reported more lifetime female partners than younger men (means: 18–24 years 6.5; 25–34 years 7.9; 35–49 years 9.5; p<0.001). In contrast, women aged 25–34 years reported more lifetime male partners than those aged 35–49 years (means: 18–24 years 2.9; 25–34 years 3.4; 35–49 years 3.1; p<0.001). For all periods (lifetime, past 5 years, past year) and all age groups, men reported more heterosexual partners than women (p<0.001).

**Table 1:** Distribution of reported numbers of heterosexual (opposite sex) partners (lifetime, past 5 years and past year) for men and women by age at interview (birth cohort): SLAHS 2000, Slovenia

No of partners	Men			All	Women			All
	Age in years (birth cohort)				Age in years (birth cohort)			
	18–24 (1975–82)	25–34 (1965–74)	35–49 (1950–64)		18–24 (1975–82)	25–34 (1965–74)	35–49 (1950–64)	
Lifetime								
0	15.3%	3.0%	1.0%	4.9%	14.6%	1.4%	0.2%	3.7%
1	16.5%	13.6%	16.5%	15.6%	31.1%	33.4%	44.9%	38.5%
2	9.6%	8.9%	10.3%	9.7%	16.2%	18.1%	14.3%	15.9%
3–4	21.8%	22.3%	19.7%	21.0%	20.1%	23.4%	20.5%	21.3%
5–9	17.5%	24.6%	20.8%	21.2%	13.0%	18.9%	13.3%	15.2%
10+	19.4%	27.7%	31.8%	27.7%	5.1%	4.8%	6.3%	5.6%
Mean* (SD)	6.5 (14.3)	7.9 (11.6)	9.5 (13.0)	8.3 (12.9)	2.9 (3.9)	3.4 (3.5)	3.1 (3.6)	3.2 (3.6)
Median (99th percentile)	3 (45)	5 (58)	5 (60)	4 (58)	2 (22)	2 (15)	2 (20)	2 (20)
Bases WT (UWT)	193 (323)	250 (186)	394 (303)	837 (812)	181 (313)	255 (214)	408 (352)	844 (879)
Past 5 years								
0	15.9%	4.8%	3.0%	6.5%	14.7%	2.8%	2.6%	5.2%
1	18.4%	52.7%	68.9%	52.6%	33.8%	76.0%	89.3%	73.5%
2	10.4%	10.1%	8.4%	9.4%	19.7%	9.7%	5.6%	9.8%
3–4	23.4%	13.1%	9.9%	14.0%	17.4%	7.0%	2.6%	7.0%
5–9	17.9%	12.6%	5.6%	10.5%	10.5%	4.5%	0.0%	3.6%
10+	13.6%	6.6%	4.2%	7.2%	3.9%	0.0%	0.0%	0.8%
Mean* (SD)	5.5 (13.3)	3.1 (4.7)	2.1 (3.1)	3.2 (7.3)	2.5 (3.4)	1.4 (1.1)	1.1 (0.5)	1.5 (1.8)
Median (99th percentile)	3 (40)	1 (30)	1 (15)	1 (30)	2 (16)	1 (5)	1 (4)	1 (8)
Bases WT (UWT)	193 (322)	249 (186)	400 (307)	842 (815)	180 (311)	258 (216)	414 (357)	851 (884)
Past year								
0	23.3%	9.3%	4.4%	10.2%	19.7%	5.3%	5.9%	8.6%
1	44.5%	70.2%	78.1%	68.1%	61.2%	89.8%	92.0%	84.8%
2	12.1%	10.9%	11.3%	11.3%	11.9%	4.1%	1.5%	4.5%
3–4	12.9%	7.3%	5.6%	7.8%	5.1%	0.9%	0.6%	1.7%
5–9	5.8%	1.8%	0.3%	2.0%	2.2%	0.0%	0.0%	0.5%
10+	1.5%	0.5%	0.3%	0.6%	0.0%	0.0%	0.0%	0.0%
Mean* (SD)	1.8 (3.7)	1.3 (1.3)	1.2 (0.8)	1.4 (2.0)	1.1 (1.0)	1.0 (0.4)	1.0 (0.3)	1.0 (0.6)
Median (99th percentile)	1 (10)	1 (7)	1 (4)	1 (8)	1 (5)	1 (2)	1 (2)	1 (3)
Bases WT (UWT)	192 (321)	248 (185)	399 (306)	840 (812)	182 (315)	259 (217)	414 (357)	855 (889)

SLAHS, Sexual Lifestyles, Attitudes and Health Survey; UWT, unweighted counts of individuals; WT, weighted counts of individuals. *Mean is not the most appropriate summary measure as the distribution is skewed. Numbers of individuals (bases) included in analyses vary according to the number of missing values for individual variables.

Table 2 shows the distribution of sexual behaviours reported by men and women by age group. Overall, 22.7% (95% CI 19.9% to 25.8%) of men and 9.5% (95% CI 7.9% to 11.4%) of women reported forming at least one new heterosexual partnership in the past year; corresponding proportions were 8.9% (95% CI 6.4% to 12.2%) of men and 1.5% (95% CI 0.8% to 3.0%) of women among those married or cohabiting and 43.1% (95% CI 38.0% to 48.4%) of men and 29.6% (95% CI 24.5% to 35.1%) of women among those single (never married) or previously married. Of all new heterosexual partnerships formed in the past year by male respondents, 83.7% were reported by those single or previously married, who together constituted 40.4% of all men. The equivalent proportions for female respondents were 88.3% and 29.0%. Mean numbers of new heterosexual partners were 0.4 for men and 0.1 for women (p<0.001). Younger individuals formed new heterosexual relationships most frequently.

**Table 2:** Distribution of reported sexual behaviours for men and women by age at interview (birth cohort): SLAHS 2000, Slovenia

Behaviours	Men			All	Women			All
	Age in years (birth cohort)				Age in years (birth cohort)			
	18–24 (1975–82)	25–34 (1965–74)	35–49 (1950–64)		18–24 (1975–82)	25–34 (1965–74)	35–49 (1950–64)	
New heterosexual* partners in past year (95% CI)								
	44.7%	21.2%	13.1%	22.7%	31.4%	5.0%	2.6%	9.5%
	39.3–50.2	16.0–27.7	9.8–17.4	19.9–25.8	26.3–36.9	2.8–9.0	1.4–5.0	7.9–11.4
Bases WT (UWT)	192 (321)	250 (187)	404 (310)	846 (818)	181 (313)	257 (216)	411 (354)	849 (883)
No of new heterosexual* partners in past year according to marital status								
Married mean (SD)	0 (0)	0.13 (0.5)	0.08 (0.3)	0.09 (0.3)	0 (0)	0 (0)	0.02 (0.2)	0.01 (0.1)
Bases WT (UWT)	3 (4)	95 (68)	300 (233)	398 (305)	10 (18)	154 (130)	340 (296)	505 (444)
Cohabiting mean (SD)	0.51 (1.7)	0.10 (0.4)	0.15 (0.4)	0.17 (0.7)	0.07 (0.3)	0.07 (0.4)	0.04 (0.2)	0.06 (0.4)
Bases WT (UWT)	13 (21)	51 (39)	39 (28)	103 (88)	25 (45)	51 (42)	26 (22)	102 (109)
Previously married† mean (SD)	–	1 (–)	1.37 (2.1)	1.32 (2.0)	–	0.75 (1.2)	0.12 (0.3)	0.20 (0.5)
Bases WT (UWT)	0 (0)	1 (1)	10 (7)	11 (8)	0 (0)	4 (3)	28 (22)	32 (25)
Single mean (SD)	1.16 (3.8)	0.62 (1.2)	0.26 (0.6)	0.87 (2.9)	0.54 (1.0)	0.23 (0.5)	0.09 (0.3)	0.43 (0.9)
Bases WT (UWT)	163 (273)	92 (71)	41 (31)	296 (375)	138 (239)	47 (40)	17 (14)	202 (293)
All mean (SD)	1.10 (3.7)	0.32 (0.9)	0.14 (0.5)	0.40 (1.9)	0.44 (0.9)	0.07 (0.3)	0.03 (0.2)	0.13 (0.5)
Bases WT (UWT)	179 (298)	239 (179)	391 (300)	809 (777)	174 (303)	256 (215)	411 (354)	841 (872)
Concurrent heterosexual* partnerships (95% CI)								
Lifetime	25.7%	30.2%	43.1%	35.3%	17.4%	15.5%	14.2%	15.3%
	21.0–31.1	23.7–37.5	37.4–48.9	31.5–39.3	13.2–22.4	11.2–21.1	10.8–18.6	12.8–18.2
Bases WT (UWT)	182 (304)	228 (170)	378 (290)	788 (764)	176 (305)	243 (205)	397 (343)	816 (853)
Past 5 years	25.6%	22.6%	25.0%	24.4%	16.4%	10.3%	3.4%	8.2%
	21.0–30.8	17.1–29.2	20.5–30.1	21.3–27.8	12.4–21.4	6.9–15.2	1.9–5.9	6.6–10.3
Bases WT (UWT)	185 (309)	241 (181)	401 (307)	827 (797)	177 (307)	255 (214)	409 (353)	841 (874)
Past year	10.7%	10.9%	16.5%	13.5%	6.4%	2.6%	1.4%	2.8%
	7.6–14.8	7.3–15.9	12.8–21.0	11.1–16.4	4.1–9.7	1.2–5.6	0.6–3.2	1.9–4.0
Bases WT (UWT)	189 (317)	248 (186)	403 (309)	841 (812)	181 (313)	257 (216)	414 (357)	852 (886)
Paid for sex with women (95% CI)								
Lifetime	3.2%	5.4%	4.5%	4.4%	–	–	–	–
	1.7–5.8	2.8–10.3	2.7–7.4	3.1–6.4	–	–	–	–
Bases WT (UWT)	193 (323)	250 (187)	392 (300)	836 (810)	–	–	–	–
Past 5 years	2.9%	3.8%	1.7%	2.6%	–	–	–	–
	1.5–5.4	1.9–7.7	0.7–4.0	1.6–4.1	–	–	–	–
Bases WT (UWT)	193 (323)	253 (189)	392 (300)	839 (812)	–	–	–	–
Had non-Slovenian heterosexual* partners (95% CI)								
Lifetime	18.9%	22.0%	27.7%	24.0%	17.9%	19.1%	24.1%	21.3%
	14.8–23.7	16.4–28.8	23.1–32.9	20.8–27.5	13.9–22.8	14.2–25.2	19.5–29.3	18.2–24.7
Bases WT (UWT)	193 (323)	254 (190)	401 (307)	848 (820)	182 (315)	256 (215)	412 (356)	850 (886)
Past 5 years	17.0%	10.4%	11.8%	12.6%	16.6%	10.9%	11.1%	12.2%
	13.2–21.7	6.6–16.1	8.4–16.3	10.1–15.6	12.7–21.5	7.1–16.4	8.0–15.2	9.8–15.1
Bases WT (UWT)	191 (320)	252 (188)	403 (309)	846 (817)	182 (315)	256 (215)	410 (354)	848 (884)
Occasions of heterosexual* sex in past 4 weeks								
0	46.7%	21.7%	14.4%	24.5%	36.0%	13.0%	14.0%	18.6%
1–4	17.2%	24.7%	28.3%	24.5%	20.0%	28.3%	34.1%	29.1%
5–9	12.9%	21.5%	28.1%	22.4%	20.1%	25.1%	30.4%	26.4%
10–29	21.3%	32.2%	29.2%	28.2%	22.9%	33.2%	21.6%	25.5%
30+	1.9%	0%	0%	0.5%	1.0%	0.5%	0%	0.4%
Mean (SD)	5.2(8.8)	6.3(5.6)	6.5 (5.3)	6.1 (6.5)	5.5 (7.6)	7.0 (5.8)	5.6 (4.6)	6.0 (5.8)
Median (99th percentile)	2 (50)	5 (20)	5 (25)	5 (25)	3 (25)	6 (20)	5 (20)	5 (20)
Bases WT (UWT)	190 (317)	243 (182)	350 (269)	783 (768)	176 (304)	242 (203)	365 (315)	783 (822)
No of occasions of heterosexual* sex in past 4 weeks								
Married/cohabiting‡ mean (SD)	14.4 (19)	7.6 (5.3)	7.3 (5.2)	7.6 (6.3)	8.7 (6.5)	7.7 (5.8)	6.1 (4.5)	6.8 (5.2)
Bases WT (UWT)	15 (25)	141 (104)	287 (222)	443 (351)	32 (57)	190 (159)	323 (281)	545 (497)
Previously married† mean (SD)	–	10 (–)	2.5 (4.0)	3.1 (4.4)	–	5.8 (5.3)	3.0 (5.2)	3.4 (5.1)
Bases WT (UWT)	0 (0)	1 (1)	15 (11)	17 (12)	0 (0)	4 (3)	26 (20)	29 (23)
Single mean (SD)	4.4 (6.8)	4.4 (5.6)	2.9 (4.6)	4.2 (6.2)	4.8 (7.6)	4.2 (4.9)	1.0 (2.4)	4.3 (6.9)
Bases WT (UWT)	175 (292)	100 (77)	48 (36)	323 (405)	143 (246)	48 (41)	17 (14)	208 (301)
All mean (SD)	5.2 (8.8)	6.3 (5.6)	6.5 (5.3)	6.1 (6.5)	5.5 (7.6)	7.0 (5.8)	5.6 (4.6)	6.0 (5.8)
Bases WT (UWT)	190 (317)	243 (182)	350 (269)	783 (768)	176 (304)	242 (203)	365 (315)	783 (822)

SLAHS, Sexual Lifestyles, Attitudes and Health Survey; UWT, unweighted counts of individuals; WT, weighted counts of individuals. *Opposite sex. †Previously married (separated, divorced or widowed). ‡Married or cohabiting. Numbers of individuals (bases) included in analyses vary according to the number of missing values for individual variables.

Concurrent partnerships at least once during their lifetime were reported by 35.3% (95% CI 31.5% to 39.3%) of men and 15.3% (95% CI 12.8% to 18.2%) of women and during the past 5 years by 24.4% (95% CI 21.3% to 27.8%) of men and 8.2% (95% CI 6.6% to 10.3%) of women. For all periods and all age groups, men consistently reported concurrency more frequently than women (p<0.05), except for 18–24 year olds for the past year (p = 0.07). As the proportion of respondents with concurrent heterosexual relationships over their lifetime is influenced by the number of sexually active years, the reporting of lifetime concurrent partners was more common in older than younger men. In contrast, women aged 18–24 years reported having ever engaged in concurrent relationships with similar frequency (17.4%) as women aged 25–34 and 35–49 years (15.5%, 14.2%, respectively).

Coercive sex was not uncommon. Women were asked “when, if ever, was the last time a man forced you into sexual intercourse” and 12.0% (95% CI 9.8% to 14.5%) of women reported having ever been forced and 4.8% during the past 5 years.

Overall, 4.4% (95% CI 3.1% to 6.4%) of men reported having ever paid for sex with a woman; 2.1% (95% CI 1.2% to 3.4%) of men reported only one occasion of paying for sex with a woman during their lifetime, but 0.9% (95% CI 0.4% to 2.0%) reported at least 10 such occasions. The mean numbers of heterosexual encounters paid for increased from 0.1 in those aged 18–24 years to 0.4 and 0.3 among older men (25–34 and 35–49 years, respectively; p = 0.03). Overall, 3.4% (95% CI 2.3% to 5.0%) of men had paid for sex with a foreign women at least once. Respondents were also asked whether they had ever received payment for sex; 0.9% (95% CI 0.4% to 2.1%) of men (no MSM) and 0.6% (95% CI 0.3% to 1.3%) of women responded that they had. Among women aged 18–24 years, 1.6% (95% CI 0.7% to 3.8%) reported receiving payment for sex, suggesting a recent increase in selling sex among the youngest (p = 0.05).

Sex with a non-Slovenian heterosexual partner during the past 5 years was reported by 12.6% (95% CI 10.1% to 15.6%) of men and 12.2% (95% CI 9.8% to 15.1%) of women. Recent non-Slovenian heterosexual partners of both men and women were most often casual (63.6%; 38.8%), followed by spouses (22.1%; 36.2%), steady partners (8.1%; 25.0%) and for men, commercial sex workers (6.2%). The last heterosexual sexual intercourse with a foreign partner occurred abroad in 48.7% of instances for men and 25.9% of instances for women, only in 5% of instances in countries outside Europe.

Men and women reported similar frequencies of heterosexual intercourse in the past 4 weeks. For both genders, the frequency of sex was lowest among the youngest age group (p<0.001). As marriage or cohabitation implies the availability of a regular sexual partner, the frequency of heterosexual intercourse was much higher in this group than in those previously married and single (p<0.001 for both genders).

Vaginal intercourse was almost universal (table 3). Lifetime oral heterosexual sex was also very common, with 79.3% (95% CI 76.2% to 82.0%) of men and 72.7% (95% CI 69.5% to 75.8%) of women reporting fellatio and 78.1% (95% CI 75.0% to 80.9%) of men and 77.3% (95% CI 74.3% to 80.1%) of women cunnilingus. Ever having had heterosexual anal intercourse was reported by 31.6% (95% CI 27.9% to 35.6%) of men and 22.3% (95% CI 19.6% to 25.4%) of women. Any occasions of heterosexual genital stimulation not resulting in intercourse (non-penetrative sex) during lifetime were reported by 77.3% (95% CI 74.0% to 80.2%) of men and 69.0% (95% CI 65.4% to 72.4%) of women. Finally, ever having had heterosexual intercourse during menstruation was reported by 45.2% (95% CI 41.7% to 48.7%) of men and 45.0% (95% CI 41.4% to 48.7%) of women.

**Table 3:** Distribution of reported heterosexual practices for men and women by age at interview (birth cohort): SLAHS 2000, Slovenia

Behaviours	Men			All	Women			All
	Age in years (birth cohort)				Age in years (birth cohort)			
	18–24 (1975–82)	25–34 (1965–74)	35–49 (1950–64)		18–24 (1975–82)	25–34 (1965–74)	35–49 (1950–64)	
Heterosexual practices ever (95% CI)								
Vaginal intercourse	84.2%	97.1%	99.1%	95.2%	85.0%	99.1%	99.8%	96.3%
	79.9–87.8	93.6–98.7	97.2–99.7	93.7–96.3	80.7–88.4	96.2–99.8	98.3–100	95.2–97.2
Bases WT (UWT)	192 (321)	257 (192)	408 (312)	857 (825)	183 (317)	256 (215)	403 (348)	842 (880)
Oral sex*	71.6%	88.1%	85.9%	83.3%	74.3%	91.0%	76.2%	80.2%
	66.4–76.4	82.6–92.1	81.4–89.5	80.4–85.8	69.3–78.8	86.1–94.3	71.2–80.5	77.2–82.9
Bases WT (UWT)	191 (319)	250 (187)	388 (297)	829 (803)	181 (314)	251 (210)	397 (343)	829 (867)
Anal intercourse	25.2%	42.4%	27.9%	31.6%	19.6%	30.6%	18.3%	22.3%
	20.3–30.9	35.1–50.0	22.9–33.5	27.9–35.6	15.6–24.4	24.7–37.2	14.6–22.6	19.6–25.4
Bases WT (UWT)	191 (319)	246 (184)	383 (294)	821 (797)	180 (312)	254 (213)	392 (339)	826 (864)
Non-penetrative sex†	75.0%	85.2%	73.2%	77.3%	69.8%	77.2%	63.4%	69.0%
	70.0–79.5	79.1–89.7	67.7–78.0	74.0–80.2	64.3–74.7	70.9–82.4	57.5–68.9	65.4–72.4
Bases WT (UWT)	193 (323)	253 (189)	386 (296)	832 (808)	183 (317)	250 (210)	395 (341)	828 (868)
Heterosexual practices past year (95% CI)								
Vaginal intercourse	77.4%	90.5%	96.0%	90.2%	79.3%	95.1%	93.6%	91.0%
	72.4–81.8	85.5–93.8	92.6–97.8	88.0–92.0	74.4–83.4	90.8–97.5	90.2–95.9	88.8–92.7
Bases WT (UWT)	192 (321)	257 (192)	408 (312)	857 (825)	183 (314)	251 (210)	497 (343)	829 (880)
Oral sex*	62.9%	82.1%	76.9%	75.3%	68.4%	81.5%	63.4%	70.0%
	57.2–68.3	76.0–86.9	71.5–81.6	71.9–78.3	63.1–73.2	75.5–86.3	58.4–68.2	66.7–73.1
Bases WT (UWT)	191 (319)	250 (187)	388 (297)	829 (803)	181 (314)	251 (210)	397 (343)	829 (867)
Anal intercourse	18.1%	24.7%	16.2%	19.2%	14.6%	16.7%	10.9%	13.5%
	14.0–23.0	18.7–31.9	12.2–21.3	16.2–22.7	11.2–18.9	12.4–22.0	8.1–14.7	11.3–16.0
Bases WT (UWT)	191 (319)	246 (184)	383 (294)	821 (797)	180 (312)	254 (213)	392 (339)	826 (864)
Non-penetrative sex†	63.8%	73.1%	58.5%	64.2%	63.1%	61.2%	46.0%	54.4%
	58.1–69.2	66.3–78.9	52.5–64.2	60.6–67.6	57.5–68.3	54.2–67.8	40.5–51.6	50.9–57.9
Bases WT (UWT)	193 (323)	253 (189)	386 (296)	832 (808)	183 (317)	250 (210)	395 (341)	828 (868)

SLAHS, Sexual Lifestyles, Attitudes and Health Survey; UWT, unweighted counts of individuals; WT, weighted counts of individuals. *Either cunnilingus or fellatio. †Genital contact only and no intercourse (penetrative sex). Numbers of individuals (bases) included in analyses vary according to the number of missing values for individual variables.

A small proportion of men (3.3%; 95% CI 2.3% to 4.8%) and women (3.6%; 95% CI 2.6% to 5.0%) reported having had a sexual experience or sexual contact (eg, kissing or cuddling) with someone of the same gender. Intercourse with someone of the same gender (oral and anal between men and oral between women) during their lifetime was reported by 1.0% (95% CI 0.5% to 2.0%) of men and 0.9% (95% CI 0.5% to 1.9%) of women. At the time of the survey, the majority of men who reported ever having had sex with men were married or cohabiting with a female partner.

Overall, 11.6% (95% CI 9.2% to 14.4%) of men and 9.1% (95% CI 7.2% to 11.5%) of women reported consistent condom use during vaginal or anal heterosexual intercourse in the past 4 weeks, and an additional 14.7% (95% CI 11.9% to 18.1%) of men and 9.9% (95% CI 7.9% to 12.3%) of women inconsistent use. Consistent and inconsistent condom use were reported more frequently among men and women who were single, separated, divorced or widowed than married or cohabiting (p<0.05) (table 4). Similarly, comparing individuals with at least two heterosexual partners in the past year with those with only one, inconsistent condom use (p<0.05) and consistent use (men p<0.05; women p = 0.09) were reported more frequently among men and women with multiple partners.

**Table 4:** Distribution of condom use during past 4 weeks for sexually active men and women by age at interview (birth cohort) and marital status: SLAHS 2000, Slovenia

	Men			All	Women			All
	Age in years (birth cohort)				Age in years (birth cohort)			
	18–24 (1975–82)	25–34 (1965–74)	35–49 (1950–64)		18–24 (1975–82)	25–34 (1965–74)	35–49 (1950–64)	
Consistent condom use (all occasions) during vaginal and anal heterosexual intercourse past 4 weeks (95% CI)								
Married or cohabiting								
	5.7%	3.0%	6.3%	5.3%	7.0%	9.7%	6.1%	7.4%
	0.8–31.3	1.0–9.1	3.8–10.4	3.4–8.3	2.5–18.0	6.1–15.3	4.0–9.4	5.5–10.0
Bases WT (UWT)	13 (21)	137 (101)	317 (244)	467 (366)	34 (60)	194 (163)	336 (293)	564 (516)
Not married or cohabiting								
	33.4%	31.6%	0%	28.0%	16.4%	23.3%	5.7%	16.3%
	26.5–41.0	20.0–46.0	–	22.4–34.4	11.5–22.9	10.7–43.3	0.8–32.0	11.2–23.0
Bases WT (UWT)	90 (151)	63 (49)	25 (19)	179 (219)	83 (142)	30 (26)	21 (17)	134 (185)
One heterosexual partner in past year								
	24.7%	9.1%	6.8%	9.7%	11.5%	11.5%	6.3%	8.7%
	17.3–33.8	5.0–16.0	4.0–11.4	7.2–13.0	7.6–17.1	7.6–17.0	4.1–9.5	6.7–11.2
Bases WT (UWT)	61 (101)	152 (114)	277 (213)	489 (428)	90 (157)	215 (181)	348 (302)	653 (640)
Two or more heterosexual partners in past year								
	39.3	16.7	1.9	16.9	22.0	12.4	0	15.5
	28.8–50.8	6.8–35.3	0.3–12.7	11.9–23.6	11.7–37.5	1.6–54.5	–	8.2–27.5
Bases WT (UWT)	41 (68)	41 (30)	60 (45)	141 (143)	25 (43)	9 (8)	9 (8)	43 (59)
All	30.0%	12.0%	5.8%	11.6%	13.7%	11.5%	6.1%	9.1%
	23.8–37.0	7.7–18.4	3.5–9.6	9.2–14.4	9.7–19.0	7.8–16.8	4.0–9.3	7.2–11.5
Bases WT (UWT)	103 (172)	200 (150)	344 (264)	647 (586)	117 (202)	224 (189)	357 (310)	698 (701)
Inconsistent condom use during vaginal and anal heterosexual intercourse in past 4 weeks (95% CI)								
Married or cohabiting								
	14.0%	16.3%	10.5%	12.3%	16.7%	11.8%	6.2%	8.8%
	4.7–34.8	10.3–24.8	7.1–15.3	9.1–16.3	9.4–27.9	7.8–17.5	4.0–9.7	6.6–11.6
Bases WT (UWT)	13 (21)	137 (101)	317 (244)	467 (366)	34 (60)	194 (163)	336 (293)	564 (516)
Not married or cohabiting								
	24.9%	20.5%	10.1%	21.2%	19.8%	10.6%	0%	14.7%
	18.8–32.1	11.2–34.7	2.5–33.3	16.0–27.6	14.0–27.3	3.4–28.2		10.3–20.5
Bases WT (UWT)	90 (151)	63 (49)	25 (19)	179 (219)	83 (142)	30 (26)	21 (17)	134 (185)
One heterosexual partner in past year								
	20.6%	14.1%	8.3%	11.6%	18.3%	11.6%	5.7%	9.4%
	13.8–29.7	8.6–22.2	5.3–12.6	8.8–15.2	13.0–25.1	7.8–16.9	3.5–9.0	7.4–11.9
Bases WT (UWT)	61 (101)	152 (114)	277 (213)	489 (428)	90 (157)	215 (181)	348 (302)	654 (640)
Two or more heterosexual partners in past year								
	26.1%	27.3%	21.7%	24.6%	21.9%	11.9%	13.7%	18.1%
	17.6–37.0	13.9–46.5	11.8–36.3	17.7–33.0	11.8–37.0	1.6–53.2	1.8–57.4	10.0–30.6
Bases WT (UWT)	41 (68)	41 (30)	60 (45)	141 (143)	25 (43)	9 (8)	9 (8)	43 (59)
All	23.5%	17.6%	10.4%	14.7%	18.9%	11.6%	5.9%	9.9%
	17.9–30.3	12.3–24.6	7.1–15.0	11.9–18.1	14.1–24.9	7.9–16.8	3.7–9.2	7.9–12.3
Bases WT (UWT)	103 (172)	200 (150)	344 (264)	647 (586)	117 (202)	224 (189)	357 (310)	698 (701)

SLAHS, Sexual Lifestyles, Attitudes and Health Survey; UWT, unweighted counts of individuals; WT, weighted counts of individuals. Numbers of individuals (bases) included in analyses vary according to the number of missing values for individual variables.

Lifetime injection of illicit drugs was reported by 0.3% (95% CI 0.1% to 1.1%) of men and 0.1% (95% CI 0.0% to 0.6%) of women and was most common among those 18–24 years old, reported by 0.7% (95% CI 0.2% to 2.6%) of men and 0.4% (95% CI 0.1% to 2.7%) of women. Individuals who injected drugs (IDU) during their lifetime reported higher numbers of lifetime heterosexual partners than non-users (means: IDU 16.4; non-IDU 5.8). All IDU reported that their most recent heterosexual partners have never injected illicit drugs. Among individuals who reported having never injected illicit drugs themselves, 1.2% (95% CI 0.7% to 2.0%) of men and 1.3% (95% CI 0.8% to 2.1%) of women reported having had a heterosexual partner in the past 5 years who had injected illicit drugs.

## DISCUSSION

Our results provide the first estimates of sexual behaviour, HIV/STI risk and recent condom use behaviours in the general population of Slovenia, and show wide variability in sexual lifestyles between individuals of different ages, men and women and according to marital status.

Our results are comparable to the British survey (NATSAL, 2000), as we used similar methods and collected the data at the same time in a probability sample of the general population, but with a slightly different age range (Slovenian 18–49 years; British 16–44 years).^14^ British people reported more lifetime heterosexual partners (means for men and women: British 12.7, 6.5; Slovenian 8.3, 3.2); during the past 5 years more men reported having sex with men (British 2.6%; Slovenian 0.6%) and having paid for sex (British 4.2%; Slovenian 2.6%), and in the past year more men and women reported new sexual partners (means: British 0.8, 0.4; Slovenian 0.4, 0.1) and more women reported concurrent heterosexual partners (British 9.0%; Slovenian 2.8%). The only HIV risk behaviour reported less frequently in the UK than in Slovenia was ever having had heterosexual anal intercourse (British men and women 12.2%, 11.3%; Slovenian men and women 31.6%, 22.3%).

**Key messages**

- Population-based data on HIV/STI risk behaviour have not previously been available in low HIV prevalence countries of central Europe.
- Recent patterns of reported sexual behaviour in a nationally representative cross-sectional survey of 18–49 year old Slovenians are consistent with a low risk of HIV/STI transmission.
- In the past 5 years, men and women reported 3.2 and 1.5 mean numbers of heterosexual partners and 24.4% and 8.2%, respectively, reported concurrent heterosexual partnerships.
- Consistent and inconsistent condom use was reported more frequently among men reporting multiple female partners and those not married or cohabiting.

Other national sexual behaviour surveys conducted in the late 1980s and 1990s in probability samples of the general populations of west European countries also reported higher mean numbers of lifetime sexual partners than our data (range 11.7–19.7 for men, 3.8–6.0 for women; Slovenian men 8.3, women 3.3); higher proportions of men who had ever paid women for sex (range 6.6–38.6%; Slovenia 4.4%) and higher proportions of men reporting at least one male sexual partner (range 2.9–11.8%; Slovenia 1.0%), with the exception of Portugal (0.9%).^15^ ^16^

Other similar surveys also reported striking gender differences in the reported numbers of heterosexual partners, which may be explained partly by social and cultural factors resulting in a tendency for men to over-report and women to under-report, by a small number of women with very high numbers of male partners and who may be underrepresented in the sample (eg, sex workers) and from age mixing, as women marry or start cohabiting at a younger age and tend to choose older partners.^14^ ^15^ ^17^^–^21

For people from low HIV and STI prevalence countries, engaging in sex with people from higher prevalence countries increases risk.^22^ ^23^ The recent increase in the reported incidence of early syphilis in Slovenia was associated with heterosexual sex in countries of the former Soviet Union with a high burden of syphilis.^24^ However, although there was quite a high rate of heterosexual sex with non-Slovenians, the great majority of such sex contacts did not occur in countries with generalised HIV epidemics.

Sex with someone of the same gender was reported rarely. As MSM have the highest HIV prevalence in Slovenia and bear a disproportionately high burden of other STI,^2^ ^3^ there is some potential for transmission to the heterosexual population, especially as the majority of MSM in our sample were married to or cohabitating with a woman at the time of the survey.

As consistent and correct use of condoms significantly reduces the risk of HIV, other STI and unplanned conception, condom use has been promoted in Slovenia as in other parts of the world. We have previously reported the steep increase over time in condom use at first heterosexual intercourse and the association with the increased likelihood of use later in sexually active life, suggesting that HIV-related condom use promotion has had an impact on preventive behaviours.^25^ Nevertheless, consistent condom use during the past 4 weeks was less prevalent in Slovenia than Britain (Slovenian men 11.6%, women 9.1%; British men 24.4%, women 18.0%). However, some evidence of the adoption of HIV/STI risk-reduction strategies with casual sex partners in Slovenia comes from the more frequent condom use during the past 4 weeks among men with at least two female partners during the past year than those with only one, as well as among those still single or previously married, which is similar to British results.^14^ The challenge remains to increase the level of condom use among Slovenians. This may not be achieved if there is a decrease in funding for condom-use promotion campaigns, as has recently occurred in other developed countries.^26^

Under 1% of Slovenian men and women aged 18–24 years reported injecting illicit drugs during the past 5 years, similar to estimates from comparable European surveys.^27^ IDU in our sample reported a higher median number of lifetime heterosexual partners than non-users, and reported that all their most recent heterosexual partners were non-injectors, indicating a potential for HIV and STI heterosexual transmission from IDU into the general population of non-users.

The methodological strengths of our survey include a reliable general population sampling frame and the use of extensively pre-tested and piloted data collection methods. The use of anonymous self-administered questionnaires may have contributed to the improved validity of self-reported information. Limitations include validity constraints on self-reported information and possible participation biases. We may have underestimated the proportions of individuals with particular higher-risk behavioural patterns that tend to be more stigmatised, for example, the proportion of MSM. However, reluctant respondents who agreed to participate only after prompting and those who responded to the first request for participation do not always show differences with respect to sexual practices.^28^ ^29^

To conclude, recent sexual behaviour, HIV/STI risk and condom use behaviour patterns indicate a low risk of HIV/STI transmission in the general population of Slovenia. Our results will inform Slovenian sexual and reproductive health policies including HIV/STI prevention, and are particularly valuable, because nationally representative population-based HIV/STI risk and sexual behaviour data have not previously been available in low HIV prevalence countries of central Europe.^21^
